# Pharmacokinetic Comparisons of Different Combinations of Yigan Jiangzhi Formula in Rats: Simultaneous Determination of Fourteen Components by UPLC-MS/MS

**DOI:** 10.1155/2020/9353975

**Published:** 2020-03-21

**Authors:** Yang Wang, Ping Wang, Jun Xie, Zhaorui Yin, Xiaoyan Lin, Yuanhong Zhao, Zheng Li, Tao Chen, Shuang Gu, Qiang Lv, Zhili Zhao, Changhua Xu

**Affiliations:** ^1^First Teaching Hospital of Tianjin University of Traditional Chinese Medicine, Tianjin 300112, China; ^2^School of Chinese Materia Medica, Tianjin University of Traditional Chinese Medicine, Tianjin 300193, China; ^3^Food Science and Technology, Shanghai Ocean University, Shanghai 201306, China; ^4^Tianjin Chase Sun Pharmaceutical Co., Ltd., Tianjin 301700, China

## Abstract

A rapid, specific, and sensitive analysis for simultaneous determination of fourteen components (daidzein, fermononetin, apigenin, luteolin, puerarin, ononin, calycosin-7-O-*β*-D-glucoside, tanshinol, rosmarinic acid, alkanoic acid, salvianolic acid B, berberine, jatrorrhizin, and palmatine) of Yigan Jiangzhi formula (YGJZF, a clinical experienced formula for damp-heat syndrome) in rat plasma was developed and validated using ultraperformance liquid chromatography coupled with mass spectrometry. Lower limit of quantitation ranged from 0.2–10.0 ng/mL, and the calibration curves showed good linearity over 500 times of measuring range. The validated method was successfully applied to the pharmacokinetics investigation of the fourteen compounds in rat plasma after oral administration of two different doses of YGJZF. Compared with the low-dose group of YGJZF, the high-dose group showed significant increase (*P* < 0.01 or *P* < 0.05) in maximum plasma concentration, maximum concentration time, and area under the plasma concentration-time curve and decrease (*P* < 0.01 or *P* < 0.05) in clearance of most of the fourteen analytes, which suggested that the bioavailability of these components could be enhanced by increasing dosage. The above results may provide useful information for cognizing the relationship between in vitro and in vivo data of the fourteen bioactive ingredients of YGJZF and further guiding rational clinical drug prescription.

## 1. Introduction

Alcoholic liver disease (ALD) is the disorder of hepatocytes structural abnormalities and (or) dysfunctions caused by long-term excessive alcohol consumption. Severe alcohol abuse can induce extensive hepatocytes necrosis and even hepatic failure [1–3]. Hence, ALD is one of the major diseases threatening human health and is also the leading cause of liver-related morbidity and mortality worldwide [4, 5] resulting in 2.5 million deaths globally each year and 1% cost of the GDP of middle- and high-income countries [6]. However, there is still no clearly therapeutic schedule that can be successfully applied to the treatment of ALD for modern medicine [2, 7].

Traditional Chinese medicine (TCM) has been used for thousands of years in clinical practice in China, especially for treating complex and chronic disorders. Generally, TCM is often used as the combination of multiple herbs to induce synergistic effects and reduce adverse reactions. As a complex system, TCM usually contains hundreds to millions of chemical components, which play the key role in its therapeutic effects [8, 9]. However, considering the perspective of pharmacokinetics (PK), only the chemical components absorbed into blood have the opportunity for exerting biological activity [10, 11].

According to the basic pathogenesis of TCM, deficiency, turbidity, and alcohol are the main starting and changing factors of ALD. Yigan Jiangzhi formula (YGJZF), a clinical experience formula (developed by Professor Yuanhong Zhao) based on Suye–Huanglian decoction (recorded in the chapter on damp heat which was written by the Qing dynasty physician Shengbai Xue), comprises *Astragalus membranaceus*, *Puerariae Lobatae* Radix, *Salvia miltiorrhiza Bge*, *Folium Perillae*, *Coptis chinensis* Franch. and *Fructus polygoni orientalis* in the ratio of 2 : 3:1 : 1.3 : 0.8 : 2, respectively, and has been used for treating ALD in the First Teaching Hospital of Tianjin University of Traditional Chinese Medicine for the attributes of ascending lucidity, descending turbidity, and resolving depression. Our previous clinical and pharmacological researches indicated that YGJZF shows definite hepatoprotective effect [12–15]. Phytochemical studies revealed various bioactive constituents of the herbal medicines in YGJZF, mainly including triterpene saponins, flavonoids, phenolic acids, tanshinones, and protoberberine-type alkaloids [16–21]. The bioactive components for hepatoprotective effect of YGJZF mainly appear in three structural types (flavonoids, phenolic acids, and protoberberine-type alkaloids), including daidzein, fermononetin, apigenin, luteolin, berberine, jatrorrhizin, palmatine, puerarin, ononin, calycosin-7-O-*β*-D-glucoside (Glc), tanshinol, rosmarinic acid, alkanoic acid, and salvianolic acid B [22–32].

PK studies on the active ingredients of TCM play an important role in predicting and evaluating the clinical efficacy and toxicity of TCMs, guiding clinical rational drug usage, and avoiding unnecessary adverse reactions [33, 34]. In clinical practice, when the patient has obvious symptoms of ALD, the oral dose of YGJZF should be increased to 2 folds of the basal rate. From the viewpoint of the correlation between PK and pharmacodynamics, we assumed that the variation of the content of active ingredients in YGJZF of different doses (in vitro and in vivo) might generate different PK characteristics and then produce different intensity of therapeutic effect on ALD. Therefore, we attempted to compare the PK profiles of daidzein, fermononetin, apigenin, luteolin, berberine, jatrorrhizin, palmatine, puerarin, ononin, calycosin-7-O-*β*-D-Glc, tanshinol, rosmarinic acid, alkanoic acid, and salvianolic acid B after oral administration of YGYZF at different dosages, which will help us to evaluate the efficacy and safety in clinical application. Until now, no data have been reported on the determination of multi-ingredients in biological samples or have compared their PK profiles after oral administration of different dosages of YGJZF. Consequently, the PK research of these components of YGJZF is necessary. And, it is essential to develop a high sensitivity and selectivity method for simultaneous determination of multiple bioactive ingredients in biological matrix for in-depth research and development of YGJZF.

In the present study, a sensitive and rapid ultraperformance liquid chromatography coupled with mass spectrometry (UPLC-MS/MS) method was developed for simultaneous determination of fourteen active components of YGJZF in rat plasma. Meanwhile, the developed approach was applied to the comparative PK profiles of the fourteen constituents in rats after oral administration of YGJZF at two doses of 13.3 g/kg and 26.6 g/kg (crude material/body weight). It is hoped that the current study would help to illuminate the action mechanism of YGJZF for treating ALD from the PK perspective.

## 2. Experimental

### 2.1. Materials and Reagents

**Figure 1:**
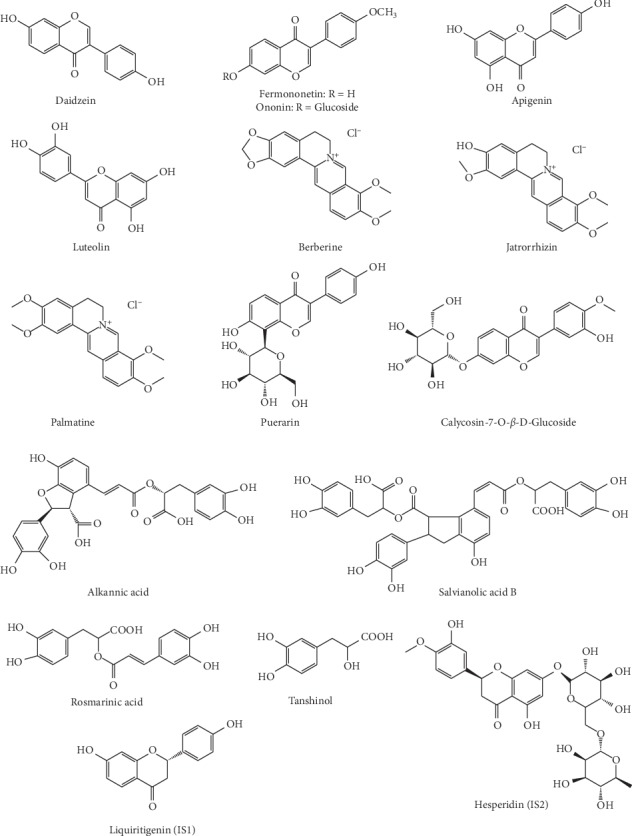
Chemical structures of fourteen analytes and two internal standards.

*Astragalus membranaceus*, *Puerariae Lobatae* Radix, *Salvia miltiorrhiza Bge*, *Folium Perillae*, *Coptis chinensis* Franch. and *Fructus polygoni orientalis* were purchased from Tianjin Traditional Chinese herbs Co. (Tianjin, China), and authenticated by Prof. Zheng Li, First Teaching Hospital of Tianjin University of Traditional Chinese Medicine. The voucher specimen was TJYGJZF 180327 (Supporting Material-Voucher specimen) and deposited at the Herbarium of Tianjin University of TCM. Luteolin, apigenin, puerarin, salvianolic acid B, calycosin-7-O-*β*-D-Glc, tanshinol, daidzein, berberine, palmatine, jatrorrhizin, liquiritigenin (IS1), and hesperidin (IS2) were purchased from the National Institute for Food and Drug Control (Beijing, China). Fermononetin, ononin, alkanoic acid, and rosmarinic acid were purchased from Chengdu Must Biotechnology (Chengdu, China). The purity of all the reference substances was higher than 98%. And, the structures of these standards are shown in Figure 1.

Acetonitrile, methanol, and formic acid (HPLC grade) were purchased from Fisher Scientific (Fair Lawn, NJ, USA). Pure water was obtained by a Milli-Q system (Millipore, Bedford, USA). All the other reagents were of analytical grade.

### 2.2. Instrument and Analytical Conditions

The UPLC-MS/MS system was a Waters Acquity™ UPLC I-Class system (Waters Corp., Milford, MA, USA) interfaced with a Waters Xevo™ TQD/MS (Waters, USA) equipped with electrospray ionization (ESI) source.

UPLC separation of the analytes and internal standards was performed on an Acquity UPLC HSS T3 column (2.1 × 100 mm, 1.8 *μ*m), and the column temperature was maintained at 35°C during the analysis. The flow rate was 0.4 mL/min, and the mobile phase consisted of 0.1% formic acid in water (A) and 0.1% formic acid in acetonitrile (B). The gradient elution conditions were as follows: 0–2 min, 95–65% A; 2–5 min, 65–45% A; 5–5.1 min, 45–2% A; 5.1–6 min, 2% A; 6–6.1 min, 2–95% A; 6.1–8 min, 95% A. The autosampler temperature was set at 4°C, and the injection volume was 6 *μ*L.

The ESI source was set in two different runs (negative or positive ionization mode) using multiple reaction monitoring (MRM) to detect the mass transitions of all analytes and internal standards. MS parameters in the source were set as follows: capillary voltage 3.0 kV (ES+), 2.7 Kv (ES−); source temperature 150°C; desolvation temperature 400°C; cone gas flow 50 L/h; desolvation gas flow 700 L/h. Cone voltage and collision energy were optimized and summarized in Table 1. All data were collected and processed by using MassLynx V4.1 workstation (Waters Corp.).

### 2.3. Preparation of Formula Sample Extract

*Astragalus membranaceus* (*180 g*), *Puerariae Lobatae* Radix (*270 g*), *Salvia miltiorrhiza Bge* (*90 g*), *Folium Perillae* (*117 g*), *Coptis chinensis* Franch. (72 g), and *Fructus polygoni orientalis* (180 g) were mixed together and then refluxed twice with water (1 : 10, w/v; 60 min each time). Dose-1: the extract was filtered through filter plate, and then the filtrates were merged and concentrated to a final concentration of 1.33 g/mL (crude material/decoction). Dose-2: the same process as dose-1, but the final concentration was at 2.66 g/mL (crude material/decoction). Contents of fourteen analytes in the two doses are shown in Table 2.

### 2.4. Preparation of Standard and Quality Control Samples

Stock solutions of fourteen analytes and two internal standards were individually prepared by dissolving 1 mg of the sixteen standard references (precisely weighed) in 1 mL of DMSO. Then, the fourteen analytes stock solutions were mixed to afford a final mixed standard solution, which contained 1 *μ*g/mL of daidzein, apigenin, berberine, jatrorrhizin, palmatine, and calycosin-7-O-*β*-D-Glc; 2.5 *μ*g/mL of fermononetin, ononin, and rosmarinic acid; 5 *μ*g/mL of luteolin and salvianolic acid B; 10 *μ*g/mL of puerarin and alkanoic acid; 50 *μ*g/mL of tanshinol. A series of mixed standard working solutions were diluted with methanol-water (50 : 50, v/v) at the ratios of 1, 2.5, 5, 10, 25, 50, 100, 250, and 500, respectively. The internal standard stock solutions were mixed and diluted with methanol-acetonitrile (50 : 50, v/v) to obtain internal standards working solution, each one with a concentration of 500 ng/mL. The stock solutions were stored in a refrigerator (4°C) until they were required for use.

Calibration standards of the analytes were prepared by spiking 100 *μ*L drug-free blank plasma with 10 *μ*L working solutions of corresponding concentrations and 300 *μ*L of internal standards working solution. The concentrations were in the range of 0.2–100 ng/mL for daidzein, apigenin, berberine, jatrorrhizin, palmatine, and calycosin-7-O-*β*-D-Glc, 0.5–250 ng/mL for fermononetin, ononin, and rosmarinic acid, 1.0–500 ng/mL for luteolin and salvianolic acid B, 2.0–1000 ng/mL for puerarin and alkanoic acid, and 10.0–5000 ng/mL for tanshinol. Three levels of quality control (QC) samples (low, medium, and high) were also prepared in the same manner. All of the above solutions were stored at −80°C before use.

### 2.5. Pretreatment of Plasma Samples

All of the plasma samples were prepared by a direct protein precipitation method with methanol-water (50 : 50, v/v). 100 *μ*L of plasma samples were spiked with 10 *μ*L of methanol-water (50 : 50, v/v) and 300 *μ*L of internal standards working solution to a 1.5 mL Eppendorf tube, vortexed for 2 min, and centrifuged at 13,000 rpm for 10 min at 4°C. The supernatant was transferred out and evaporated to dryness under a flow of gentle nitrogen gas at 40°C. Then, the residue was reconstituted with 100 *μ*L of methanol-water (50 : 50, v/v), vortexed for 2 min, and centrifuged at 13,000 rpm for 10 min at 4°C. Finally, the supernatant was transferred into autosampler vials, and 6 *μ*L of it was injected into the UPLC–MS/MS system for analysis.

### 2.6. Method Validation

The analytical method was validated with specificity, linearity, lower limit of quantification (LLOQ), precision, accuracy, extraction recovery, matrix effect, and stability in rat plasma according to the Guidance for Industry Bioanalytical Method Validation [35].

#### 2.6.1. Specificity

Selectivity was evaluated by comparing different chromatograms of drug-free blank plasma, drug-free blank plasma spiked with fourteen analytes and two internal standards, and the plasma samples from the rats after oral administration of YGJZF at different dosages, respectively.

#### 2.6.2. Linearity and Lower Limits of Quantification

The linearity of the calibration curves of all analytes was generated by plotting the peak area ratio (*y*) of each analyte to the corresponding internal standard for each standard solution versus the nominal concentration (*x*, ng/mL) of the calibration standard, with weighted (1/*x*^2^) least square linear regression. The linearity was evaluated by means of correlation coefficient (*r*) of each calibration curve. IS1 was used as internal standard for positive ion mode detection (daidzein, apigenin, berberine, jatrorrhizin, palmatine, calycosin-7-O-*β*-D-Glc, fermononetin, ononin, luteolin, and puerarin) and IS2 for the negative ion mode (rosmarinic acid, salvianolic acid B, alkanoic acid, and tanshinol), respectively. The lower limit of quantification (LLOQ) was defined as the lowest concentration on the calibration curve that can be quantitated with accuracy and precision less than 20%. And, the LLOQ was determined as at least 10 times of the signal/noise ratio.

#### 2.6.3. Precision and Accuracy

The intraday precision (evaluated with the relative standard deviation, RSD) and accuracy (evaluated with the relative error, RE) were assessed in a single day by using six replicates of QC samples at three concentrations (low, medium, and high). And, the interday precision and accuracy were determined by employing the QC samples on three consecutive days. The RSD and RE were calculated using the following equations:

(1) $$\begin{array}{c} \text{RSD}=\frac{\text{standard deviation}\left(\text{SD}\right)}{\text{mean measured concentration }}\times 100\%,\\ \text{RE}=\frac{\mathrm{mean}\mathrm{measured}\mathrm{concentration}-\mathrm{nominal}\mathrm{concentration}}{\mathrm{nominal}\mathrm{concentration}}\times 100\%. \end{array}$$

The value of RSD and RE should be less than 15%. And, the concentrations of QC samples were calculated by the calibration curve of freshly prepared samples.

#### 2.6.4. Extraction Recovery and Matrix Effect

The extraction recoveries and matrix effects of all analytes were determined by assaying QC samples at three concentrations (*n* = 6, each concentration). The extraction recoveries were calculated by comparing the peak areas of the extracted QC samples (set A) with postextraction blank plasma spiked with working standard solutions in corresponding concentrations at three levels of QC samples (set B). By comparing the peak areas acquired from set B with the pure standard solutions in corresponding concentrations at three levels of QC samples (set C), the matrix effects were evaluated. Moreover, extraction recoveries and matrix effects of the two internal standards were evaluated by the same method at one concentration (50 ng/mL).

#### 2.6.5. Stability

The stability of the fourteen analytes was determined by using six replicates of QC samples at three concentrations in different conditions: short-term stability at room temperature (25°C) for 4 h, postpreparative stability in treated plasma samples in autosampler (4°C) for 24 h, long term stability at three freeze-thaw cycles (−80°C to 25°C as one cycle), and storage at −80°C for 30 days.

### 2.7. Pharmacokinetics Study

Twelve male Sprague-Dawley rats (200–230 g body weight) were supplied by the Beijing Vital River Laboratory Animal Technology Co., Ltd (Beijing, China). All of the animals were kept under controlled laboratory condition (at 23 ± 2°C, with relative humidity of 60 ± 5% and on a 12 h dark-light cycle) for 7 days with free access to standard diet and water. All of the rats were fasted for 12 h with free access to water prior to the oral administration of the YGJZF extracts. The animal study was carried out according to the Guidelines for the Care and Use of Laboratory Animals of the First Teaching Hospital of Tianjin University of Traditional Chinese Medicine. The twelve rats were randomly divided into two groups (*n* = 6, per group) and oral administered with single dose-1 (13.3 g/kg) or dose-2 (26.6 g/kg), respectively. Blood samples (0.5 mL) of the rats were collected from the fossa orbitalis vein at 0, 0.083, 0.17, 0.33, 0.5, 0.75, 1, 2, 3, 5, 7, 9, 12, 24, 36, 48, and 72 h after dosing into heparinized 1.5 mL Eppendorf tube and centrifuged at 6000 rpm for 10 min. Then, the supernatant was immediately transferred and stored at −80°C until analysis.

PK parameters, including maximum plasma concentration (*C*_max_), maximum concentration time (*T*_max_), terminal half-life value (*T*_1/2_), area under the plasma concentration-time curve from 0 to infinity (AUC_0−∞_), area under the plasma concentration-time curve from 0 to the last time (AUC_0−*t*_), mean residence time (MRT), and clearance (CL), were calculated by using DAS 3.0 software (Mathematical Pharmacology Professional Committee of China, Shanghai, China). Statistical analysis comparisons of these PK parameters between two groups were performed by SPSS (version 19.0, Armonk, NY, USA), and a *P* < 0.05 was considered statistically significant. All data were presented as mean ± SD.

## 3. Results and Discussion

### 3.1. Optimization of UPLC-MS/MS Conditions

To obtain satisfactory chromatographic behaviors for all analytes and internal standards, two columns were examined: ACQUITY UPLC® HSS T3 (2.1 × 100 mm, 1.8 *μ*m; Waters Corp., Wexford, Ireland) and ACQUITY UPLC® BEH C18 (2.1 × 100 mm, 1.7 *μ*m; Waters Corp., Wexford, Ireland). The T3 column was selected as it could provide efficient chromatographic separation. Different mobile phase compositions (acetonitrile-water and methanol-water) were investigated for achieving better elutive power and lower background noise. And, it was observed that the former contains better effect. Meanwhile, by comparing different concentrations (0.05%, 0.1%, and 0.2%) of polarity regulators (formic acid and acetic acid) in mobile phase, conclusion was formed that adding 0.1% formic acid to the mobile phase system could improve the peak shape and enhance the resolution and sensitivity of the analytes and internal standards. Therefore, acetonitrile-water (adding 0.1% formic acid) was employed as the mobile phase for our present study. In order to optimize the precursor and product ions of all analytes and internal standards, solutions of these compounds at a concentration 400 ng/mL were directly infused into the mass spectrometer.

**Figure 2:**
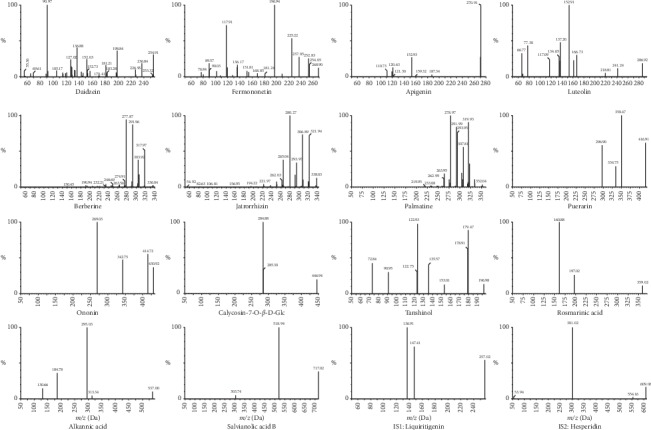
Product ion mass spectra of fourteen analytes and two internal standards.

The ESI sources were set in two different runs (negative or positive ionization modes). Daidzein, apigenin, berberine, jatrorrhizin, palmatine, calycosin-7-O-*β*-D-Glc, fermononetin, ononin, luteolin, and puerarin responded perfectly in the positive ion mode; in contrast, the others were stable and exhibited higher abundance in the negative ion mode. In order to ensure the accuracy of quantitative, two compounds (liquiritigenin (IS1) for positive and hesperidin (IS2) for the negative ion mode) were selected as the internal standards for calculating the concentration of corresponding analytes. In the full scan mode, the protonated molecular ion [M + H]^+^ of the analytes detected in the positive mode showed higher abundance and stability, whereas the deprotonated molecule [M − H]^−^ of the remaining analytes was the preponderant ion in the negative mode. Under the product ion scan mode, the structurally similar molecule may have the same fragment pathway. For instance, ononin and calycosin-7-O-*β*-D-Glc are two flavonoid glycosides; in collision chamber, they easily break their glycosidic bonds and both produced the fragment ion of [M + H-C_6_H_10_O_5_]^+^ at *m*/*z* 269.0 and 284.9. Moreover, berberine and jatrorrhizin (as two typical protoberberine-type alkaloids) produced the identical ion of [M + H-C_3_H_6_O]^+^ at *m*/*z* 277.9 and 280.3, while the other two phenolic acids of rosmarinic acid and salvianolic acid B both displayed the ion of [M-H-C_9_H_10_O_5_]^−^ at 160.9 and 518.9. In addition, it was interesting to note that apigenin and luteolin (two semblable flavonoid aglycones) had the same product ion at *m*/*z* 152.9, which was corresponding to the different fragment ions of [M + H-C_8_H_6_O]^+^ and [M + H-C_8_H_6_O_2_]^+^, respectively. And, the most abundant and stable product ions of daidzein, fermononetin, palmatine, puerarin, tanshinol, alkanoic acid, IS1, and IS2 were [M + H-C_9_H_8_O_3_]^+^ (*m*/*z* 91.0), [M + H-C_3_H_4_O_2_]^+^ (*m*/*z* 196.9), [M + H-C_3_H_5_O_2_]^+^ (*m*/*z* 279.0), [M + H-C_4_H_8_O_4_]^+^ (*m*/*z* 296.9), [M-H-C_2_H_2_O_3_]^−^ (*m*/*z* 122.9), [M-H-C_10_H_10_O_7_]^−^ (*m*/*z* 295.0), [M + H-C_8_H_8_O]^+^ (*m*/*z* 136.9), and [M-H-C_12_H_20_O_9_]^−^ (*m*/*z* 301.0), separately. The optimized operating parameters for the analytes and internal standards are summarized in Table 1, and the product spectra of the sixteen compounds are shown in Figure 2.

### 3.2. Optimization of Sample Preparation

Sample preparation plays an important role in ensuring the accuracy and reliability of in vivo quantitative results. Different methods of liquid-liquid extraction and protein precipitation were tested for comparing the extraction capability. The result showed that the recovery of phenolic acids is relatively low when using liquid-liquid extraction (solvents containing ethyl acetate or *n*-butanol). Protein precipitation is simple and easy to operate despite the resulting precipitation is heterogeneous and incomplete while acetonitrile or methanol was used alone. In conclusion, a simple one-step plasma protein-precipitating procedure with methanol-acetonitrile (50 : 50, v/v) was carried out for sample preparation, which could afford higher recovery and better precision for the analytes and internal standards.

### 3.3. Method Validation

#### 3.3.1. Specificity

**Figure 3:**
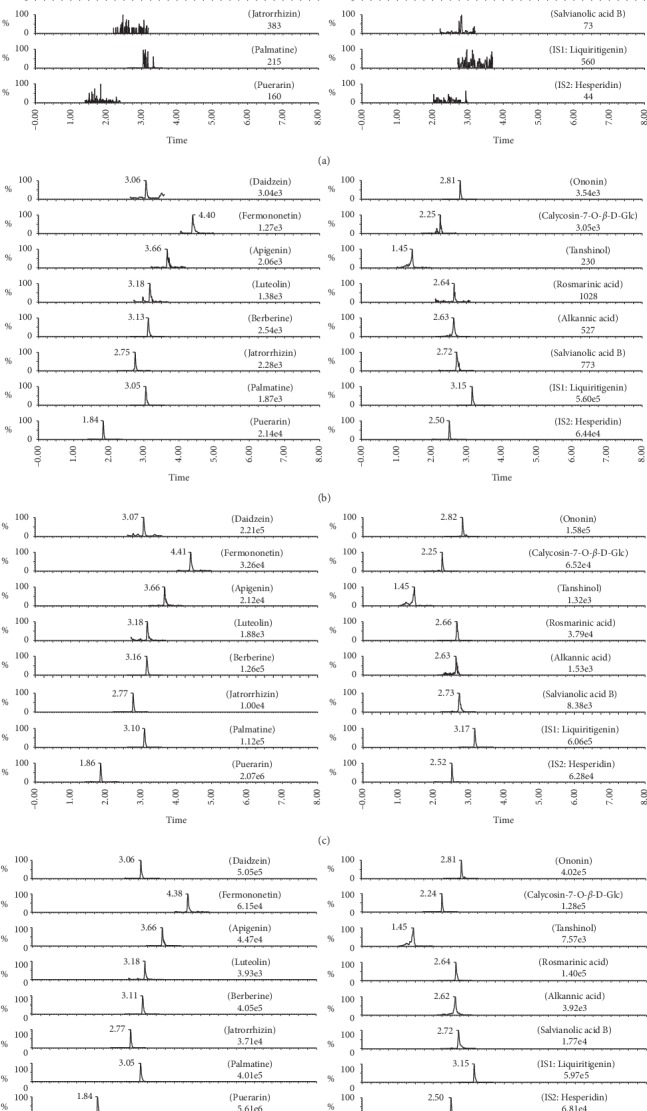
Representative MRM chromatograms of (a) blank plasma, (b) blank plasma spiked with the analytes at LLOQ and internal standards, (c) plasma sample after oral administration of dose-1 for 0.5 h, and (d) plasma sample after oral administration of dose-2 for 0.5 h.

The representative chromatograms of blank plasma, blank plasma spiked with fourteen analytes, and two internal standards and plasma samples at 0.5 h (after oral administration of different dosages of YGJZF) are shown in Figures 3(a)–3(d). It is easy to find that all analytes and two internal standards were eluted out within 5 min. And, the background was very low, and no interference from endogenous substances and metabolites was observed.

#### 3.3.2. Linearity and Lower Limits of Quantification

The calibration curves, correlation coefficients, linear ranges, and LLOQs of all analytes are shown in Table 3. All calibration curves showed good linearity (*r* ≥ 0.9933).

#### 3.3.3. Precision and Accuracy

The precision and accuracy of the intra- and interday variation of the fourteen analytes are shown in Table 4. RSD values of precision were in the range of 1.36–13.60%, and the RE of accuracy was within 11%.

#### 3.3.4. Extraction Recovery and Matrix Effect

The extraction recoveries and matrix effects of the fourteen analytes were evaluated by QC samples at three concentration levels (Table 5). The average extraction recoveries of all analytes and internal standards were in the range of 74.65–108.92%. And, the mean matrix effects were between 71.00% and 114.98%, and no obvious matrix effects were observed from analytes and internal standards. Meanwhile, the variations (RSD) of extraction recoveries and matrix effects of the same analytes at three QC concentration levels were within 12%, demonstrating the consistence in these data.

#### 3.3.5. Stability

The stability of the fourteen analytes in rat plasma under different conditions is investigated and summarized in Table 6. RE and RSD values of these analytes were in the range of −7.67–6.39 and 1.02–13.92%. It was demonstrated that the analytes in rat plasma were stable after storage at room temperature for 4 h, at 4°C (in autosampler) for 24 h, three freeze-thaw cycles, and at −80°C for 30 days.

### 3.4. Pharmacokinetics Study

**Figure 4:**
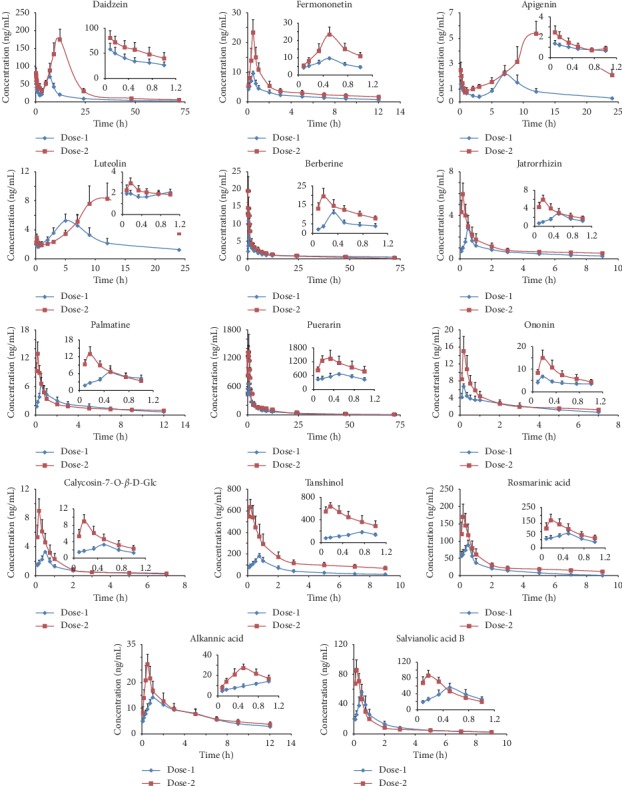
Concentration-time curves of the fourteen analytes in rat plasma after oral administration of dose-1 and dose-2 of YGJZF (mean ± SD, *n* = 6).

A comparative PK investigation of the fourteen constituents in Sprague-Dawley rats after oral administration of two doses of YGJZF was conducted with the validated method. The mean plasma concentration-time profiles of the fourteen analytes are shown in Figure 4, and the main PK parameters (*C*_max_, *T*_max_, *t*_1/2_, AUC_0−*t*_, AUC_0−∞_, MRT_0−*t*_ and CL) are listed in Table 7.

*C*_max_ and *T*_max_ are primarily used to describe the absorption rate of drugs in the body. As the data showed, most of the fourteen analytes could reach *C*_max_ in an hour after administration. Compared with those of the dose-1, nine analytes in dose-2, including berberine, jatrorrhizin, palmatine, puerarin, calycosin-7-O-*β*-D-Glc, tanshinol, rosmarinic acid, alkanoic acid, and salvianolic acid B exhibited a relatively short *T*_max_, indicating that these compounds were absorbed into blood more quickly after applying higher dose. The remaining compounds were all flavonoid glycosides or aglycones, in which *T*_max_ of daidzein, apigenin, and luteolin was delayed; however, there was no change in that of fermononetin and ononin. Meanwhile, a bimodal phenomenon of 3 flavonoids (daidzein, apigenin, and luteolin) was presented in Figure 4. In accordance with what had been reported previously [36–38], the first peak appeared in less than 1 h and the second peak at about 5 h or even later probably due to distribution, reabsorption, and enterohepatic circulation [39–41].

Upon the dose-2 level, most of the analytes showed significantly higher value (*P* < 0.01 or *P* < 0.05) in *C*_max_ and AUC, and among them, apigenin and tanshinol were increased nearly four times can be observed, suggesting the absorption of these analytes could be significantly improved after administration of dose-2. One could find that the contents of salvianolic acid B were higher than those of tanshinol in YGJZF extracts (Table 2), but in vivo, the opposite was true (Table 7). This phenomenon is consistent with the previous literature reports [42, 43]. In rats' plasma, besides the prototypical-tanshinol, these also include the tanshinol which was metabolized by salvianolic acid B [44]. In addition, as shown in Table 7, compared with dose-1, the CL of tanshinol and apigenin in dose-2 was significantly decreased (*P* < 0.01).

The concentration of berberine, jatrorrhizin, and palmatine had a distinct decline after oral administration (Figure 4, Tables 2 and 7), which indicated that the oral bioavailability of these alkaloids was very poor due to its structure and other reasons [45]. Moreover, Table 7 shows the MRT_0−*t*_ of berberine in dose-2 was significantly decreased (*P* < 0.05). The above evidence demonstrating that the CL of most of the analytes was slowed down and the action time in vivo was prolonged by increasing the dosage. Furthermore, compared with the dose-1, the dose-2 showed significantly longer (*P* < 0.01 or *P* < 0.05) in *t*_1/2_ of apigenin, luteolin, jatrorrhizin, palmatine, calycosin-7-O-*β*-D-Glc, tanshinol, rosmarinic acid, alkanoic acid, and salvianolic acid B. It is suggested that, as the dose increased, the elimination of the components slowed down.

Based upon these results, increasing the dosage enhanced the absorption, improved the bioavailability, and delayed the elimination of the most of fourteen bioactive components in YGJZF. The findings described in the present study would be meaningful for explaining why the dose-2 has better clinical efficacy in treatment of severe ALD than that of dose-1, and which is also informative for future application of YGJZF in clinical practice. Furthermore, the intensive study on toxicology of YGJZF will be involved in the following research to avoid side effects as well as to ensure clinical efficacy.

## 4. Conclusion

A rapid, accurate, and sensitive UPLC-MS/MS method for simultaneous determination of fourteen bioactive components in rat plasma was developed and successfully applied to the comparative PK study after oral administration of YGJZF at two different doses (13.3 g/kg and 26.6 g/kg). It is the first time to simultaneously determine these multiple components in rat plasma after oral administration of YGJZF. By comparing the PK parameters of components in the two dosing groups, the in vivo process of YGJZF has been intuitively presented through a digital way. The obtained results demonstrated that systemic exposure level of most of the fourteen components was increased and actuation duration was prolonged in a dose-dependent manner, which could improve their bioavailability. Therefore, we speculated that the changes in PK profiles of the two doses of YGJZF might be the primary reason for their difference of the therapeutic intensity on ALD. Our comprehensive PK information will be useful for explaining the essence of clinical treatment and rational drug use of YGJZF.

## Figures and Tables

**Table 1:** Optimized MS/MS transitions, parameters, and retention time of the analytes and internal standards.

No.	Analytes	Ion mode	Parent (*m*/*z*)	Daughter (*m*/*z*)	Cone (V)	Collision (eV)	Retention time (min)
1	Daidzein	ES+	254.9	91.0	52	40	3.06
2	Fermononetin	ES+	268.9	196.9	56	40	4.37
3	Apigenin	ES+	270.9	152.9	62	30	3.68
4	Luteolin	ES+	286.9	152.9	66	32	3.18
5	Berberine	ES+	336.0	277.9	12	44	3.12
6	Jatrorrhizin	ES+	338.0	280.3	52	40	2.75
7	Palmatine	ES+	352.0	279.0	54	54	3.05
8	Puerarin	ES+	416.9	296.9	46	24	1.84
9	Ononin	ES+	430.9	269.0	28	16	2.81
10	Calycosin-7-O-*β*-D-Glc	ES+	446.9	284.9	32	24	2.25
11	Tanshinol	ES−	197.0	122.9	24	20	1.44
12	Rosmarinic acid	ES−	359.0	160.9	32	18	2.64
13	Alkanoic acid	ES−	537.0	295.0	20	22	2.62
14	Salvianolic acid B	ES−	717.0	518.9	38	20	2.72
IS1	Liquiritigenin	ES+	257.0	136.9	38	24	3.15
IS2	Hesperidin	ES−	609.1	301.0	46	24	2.50

**Table 2:** The quantitative results of the fourteen analytes in dose-1 and dose-2 of YGJZF.

No.	Analyte	Concentration (ng/mL)	
		Dose-1	Dose-2
1	Daidzein	103750	225000
2	Fermononetin	11250	23200
3	Apigenin	2250	6550
4	Luteolin	5500	12000
5	Berberine	607500	1015000
6	Jatrorrhizin	106250	170000
7	Palmatine	172500	270000
8	Puerarin	12697500	16910000
9	Ononin	116250	210000
10	Calycosin-7-O-*β*-D-Glc	90000	140000
11	Tanshinol	620000	975000
12	Rosmarinic acid	950000	1230000
13	Alkanoic acid	146250	245000
14	Salvianolic acid B	1346250	1595000

**Table 3:** The regression equation, linear ranges, and LLOQs of the fourteen analytes in rat plasma.

No.	Analyte	Regression equation (*W* = 1/*x*^2^)	*r*	Linear range (ng/mL)	LLOQ (ng/mL)
1	Daidzein	*y* = 1.0767*x* + 2.3433	0.9933	0.2–100	0.2
2	Fermononetin	*y* = 0.1497*x* + 0.0763	0.9981	0.5–250	0.5
3	Apigenin	*y* = 0.5257*x* + 0.1422	0.9975	0.2–100	0.2
4	Luteolin	*y* = 0.2124*x* + 0.0092	0.9992	1.0–500	1.0
5	Berberine	*y* = 1.0672*x* + 0.7246	0.9979	0.2–100	0.2
6	Jatrorrhizin	*y* = 1.9106*x* + 0.3999	0.9985	0.2–100	0.2
7	Palmatine	*y* = 2.5077*x* + 0.4315	0.9993	0.2–100	0.2
8	Puerarin	*y* = 0.1864*x* + 2.7353	0.9937	2.0–1000	2.0
9	Ononin	*y* = 1.7778*x* + 0.1393	0.9980	0.5–250	0.5
10	Calycosin-7-O-*β*-D-Glc	*y* = 0.9216*x* + 0.1215	0.9989	0.2–100	0.2
11	Tanshinol	*y* = 0.0158*x* − 0.0041	0.9979	10.0–5000	10.0
12	Rosmarinic acid	*y* = 0.9884*x* + 0.0390	0.9943	0.5–250	0.5
13	Alkanoic acid	*y* = 0.1800*x* + 0.0130	0.9989	2.0–1000	2.0
14	Salvianolic acid B	*y* = 0.4326*x* + 0.0395	0.9987	1.0–500	1.0

**Table 4:** Accuracy and precision of the fourteen analytes in rat plasma (mean ± SD, *n* = 3 days, 6 replicates per day).

No.	Analyte	Conc. (ng/mL)	Intraday			Interday		
			Measured (ng/mL)	Acc. (%)	Prec. (%)	Measured (ng/mL)	Acc. (%)	Prec. (%)
1	Daidzein	0.50	0.48 ± 0.04	−4.00	8.33	0.48 ± 0.04	−4.00	8.33
		10.00	10.03 ± 1.07	0.30	10.67	10.56 ± 0.75	5.60	7.10
		80.00	81.32 ± 1.59	1.65	1.96	83.14 ± 2.17	3.93	2.61
2	Fermononetin	1.25	1.32 ± 0.04	5.60	3.03	1.22 ± 0.12	−2.40	9.84
		25.00	26.12 ± 0.95	4.48	3.64	26.27 ± 0.88	5.08	3.35
		200.00	209.67 ± 5.14	4.83	2.45	214.35 ± 8.29	7.18	3.87
3	Apigenin	0.5	0.53 ± 0.05	6.00	9.43	0.51 ± 0.05	2.00	9.80
		10	10.13 ± 0.32	1.30	3.16	9.86 ± 0.37	−1.40	3.75
		80	82.42 ± 2.20	3.03	2.45	80.41 ± 3.39	0.51	4.22
4	Luteolin	2.5	2.65 ± 0.09	6.00	3.40	2.51 ± 0.27	0.40	10.76
		50	52.03 ± 1.42	4.06	2.73	52.18 ± 1.56	4.36	2.99
		400	426.65 ± 8.57	6.66	2.01	408.59 ± 16.33	2.15	4.00
5	Berberine	0.5	0.48 ± 0.04	−4.00	8.33	0.49 ± 0.06	−2.00	12.24
		10	10.83 ± 0.34	8.30	3.14	10.59 ± 0.92	5.80	8.69
		80	86.55 ± 1.31	8.19	1.51	83.71 ± 3.11	4.64	3.72
6	Jatrorrhizin	0.5	0.53 ± 0.04	6.00	7.55	0.52 ± 0.05	4.00	9.62
		10	10.68 ± 0.19	6.80	1.78	10.95 ± 0.52	9.50	4.75
		80	77.22 ± 1.67	−3.48	2.16	77.54 ± 1.97	−3.07	2.54
7	Palmatine	0.5	0.52 ± 0.04	4.00	7.69	0.55 ± 0.05	10.00	9.09
		10	10.37 ± 0.23	3.70	2.22	10.54 ± 0.62	5.40	5.88
		80	79.30 ± 1.08	−0.88	1.36	80.02 ± 3.02	0.02	3.77
8	Puerarin	5	5.11 ± 0.47	2.20	9.20	5.23 ± 0.68	4.60	13.60
		100	102.31 ± 7.76	2.31	7.58	104.50 ± 6.69	4.50	6.40
		800	778.29 ± 16.52	−2.71	2.12	782.42 ± 18.26	−2.20	2.33
9	Ononin	1.25	1.27 ± 0.08	1.60	6.30	1.28 ± 0.11	2.40	8.59
		25	25.87 ± 0.76	3.48	2.94	25.70 ± 0.62	2.80	2.41
		200	185.75 ± 5.04	−7.13	2.71	178.21 ± 7.76	−10.90	4.35
10	Calycosin-7-O-*β*-D-Glc	0.5	0.48 ± 0.04	−4.00	8.33	0.51 ± 0.04	2.00	7.84
		10	10.03 ± 0.18	0.30	1.79	9.65 ± 0.46	−3.50	4.77
		80	80.43 ± 1.38	0.54	1.72	74.57 ± 5.46	−6.79	7.32
11	Tanshinol	25	26.35 ± 2.71	5.40	10.28	24.63 ± 2.26	−1.48	9.18
		500	506.70 ± 13.32	1.34	2.63	475.29 ± 30.29	−4.94	6.37
		4000	3786.47 ± 111.61	−5.34	2.95	3691.07 ± 146.59	−7.72	3.97
12	Rosmarinic acid	1.25	1.18 ± 0.08	−5.60	6.78	1.20 ± 0.07	−4.00	5.83
		25	23.63 ± 0.57	−5.48	2.41	23.05 ± 1.06	−7.80	4.60
		200	220.90 ± 4.96	10.45	2.25	215.64 ± 12.18	7.82	5.65
13	Alkanoic acid	5	5.02 ± 0.55	0.40	10.96	4.79 ± 0.49	−4.20	10.23
		100	100.22 ± 2.23	0.22	2.23	94.08 ± 5.46	−5.92	5.80
		800	758.75 ± 26.20	−5.16	3.45	749.11 ± 22.27	−6.36	2.97
14	Salvianolic acid B	2.5	2.72 ± 0.08	8.80	2.94	2.44 ± 0.25	−2.40	10.25
		50	52.00 ± 0.82	4.00	1.58	48.57 ± 3.18	−2.86	6.55
		400	406.38 ± 5.96	1.60	1.47	404.17 ± 14.87	1.04	3.68

**Table 5:** Extraction recovery and matrix effect of the fourteen analytes and two internal standards in rat plasma (mean ± SD, *n* = 6).

No.	Analyte	Conc. (ng/mL)	Extraction recovery	RSD (%)	Matrix effect	RSD (%)
1	Daidzein	0.5	98.11 ± 5.64	5.75	106.77 ± 8.84	8.28
		10	105.24 ± 7.57	7.19	103.41 ± 6.04	5.84
		80	96.40 ± 2.69	2.79	99.75 ± 2.65	2.66
2	Fermononetin	1.25	88.58 ± 4.13	4.66	96.12 ± 7.71	8.02
		25	90.70 ± 6.63	7.31	92.17 ± 5.20	5.64
		200	91.50 ± 3.22	3.52	94.97 ± 8.94	9.41
3	Apigenin	0.5	94.69 ± 5.12	5.41	90.62 ± 7.89	8.71
		10	102.92 ± 4.14	4.02	94.48 ± 1.69	1.79
		80	87.33 ± 2.47	2.83	98.42 ± 2.46	2.50
4	Luteolin	2.5	74.65 ± 8.93	11.96	94.35 ± 4.82	5.11
		50	77.32 ± 3.36	4.35	94.32 ± 4.98	5.28
		400	74.67 ± 0.94	1.26	92.93 ± 1.17	1.26
5	Berberine	0.5	99.45 ± 4.34	4.37	92.95 ± 8.82	9.49
		10	96.10 ± 3.97	4.13	89.92 ± 2.91	3.23
		80	97.45 ± 3.43	3.52	88.10 ± 1.24	1.41
6	Jatrorrhizin	0.5	108.92 ± 3.05	2.80	89.39 ± 2.11	2.36
		10	97.06 ± 3.79	3.91	80.54 ± 1.37	1.70
		80	96.54 ± 1.93	2.00	93.94 ± 1.79	1.91
7	Palmatine	0.5	92.76 ± 4.93	5.31	101.12 ± 4.91	4.86
		10	97.03 ± 5.38	5.54	91.39 ± 3.63	3.97
		80	99.60 ± 3.04	3.05	92.68 ± 1.57	1.69
8	Puerarin	5	94.45 ± 7.51	7.95	97.14 ± 4.81	4.95
		100	93.55 ± 5.46	5.84	106.56 ± 1.87	1.75
		800	98.28 ± 2.68	2.73	89.56 ± 1.80	2.01
9	Ononin	1.25	104.03 ± 6.38	6.13	82.93 ± 2.29	2.76
		25	104.11 ± 2.28	2.19	81.66 ± 1.16	1.41
		200	98.41 ± 1.38	1.40	89.79 ± 2.29	2.55
10	Calycosin-7-O-*β*-D-Glc	0.5	98.21 ± 4.75	4.84	71.00 ± 4.58	6.45
		10	104.26 ± 2.43	2.33	73.04 ± 4.41	6.04
		80	96.91 ± 3.98	4.10	77.70 ± 1.51	1.94
11	Tanshinol	25	98.86 ± 6.14	6.21	98.67 ± 1.80	1.82
		500	97.99 ± 3.65	3.72	90.88 ± 2.48	2.73
		4000	102.47 ± 2.03	1.98	87.06 ± 4.37	5.02
12	Rosmarinic acid	1.25	95.45 ± 4.53	4.75	94.79 ± 9.09	9.59
		25	96.60 ± 2.76	2.86	96.08 ± 6.78	7.06
		200	96.92 ± 3.84	3.96	96.80 ± 2.84	2.93
13	Alkanoic acid	5	88.12 ± 7.80	8.85	111.53 ± 8.22	7.37
		100	86.99 ± 4.42	5.08	112.35 ± 6.11	5.44
		800	92.60 ± 2.20	2.38	106.26 ± 2.43	2.29
14	Salvianolic acid B	2.5	85.57 ± 9.14	10.68	111.95 ± 8.63	7.71
		50	83.19 ± 3.37	4.05	114.98 ± 9.97	8.67
		400	83.81 ± 4.66	5.56	112.75 ± 2.83	2.51
IS1	Liquiritigenin	50	93.46	6.18	98.20	4.35
IS2	Hesperidin	50	89.27	3.88	100.79	4.70

**Table 6:** Stability of the fourteen analytes in rat plasma under different conditions (mean ± SD, *n* = 6).

No.	Analyte	Conc. (ng/mL)	25°C for 4 h		4°C for 24 h		Three freeze-thaw cycles		−80°C frozen for 30 days	
			Acc. (%)	Prec. (%)	Acc. (%)	Prec. (%)	Acc. (%)	Prec. (%)	Acc. (%)	Prec. (%)
1	Daidzein	0.5	4.78	4.63	−3.75	6.67	2.59	2.96	3.27	3.46
		10	1.57	2.49	1.43	7.78	−1.60	6.25	1.69	5.90
		80	−4.09	6.11	2.46	8.30	−2.36	9.06	2.35	8.54
2	Fermononetin	1.25	−2.79	3.63	1.35	7.52	2.94	4.82	−3.15	1.25
		25	2.88	6.10	3.09	4.52	2.09	7.39	1.32	5.28
		200	2.67	4.54	−2.81	5.65	4.20	7.94	5.25	1.87
3	Apigenin	0.5	4.04	6.67	1.58	4.49	0.96	2.17	3.06	6.87
		10	−2.43	4.84	−2.84	4.45	−1.60	6.25	3.25	2.38
		80	−3.10	3.15	−3.77	3.60	−5.06	8.28	4.68	3.26
4	Luteolin	2.5	1.77	6.67	−1.33	1.68	1.33	4.67	−2.16	3.76
		50	2.90	4.85	0.71	2.12	6.25	2.19	4.42	3.67
		400	1.44	3.00	1.26	4.10	1.37	2.06	1.88	2.74
5	Berberine	0.5	−3.49	6.67	3.03	6.25	4.16	5.05	2.79	5.61
		10	2.57	4.58	−2.42	10.02	−3.63	4.27	2.11	3.38
		80	2.44	4.19	−2.60	3.98	−4.35	3.03	3.70	5.49
6	Jatrorrhizin	0.5	2.22	2.78	−3.67	6.67	3.33	5.04	6.39	8.25
		10	−1.10	1.99	0.57	2.48	2.56	3.21	−3.73	4.81
		80	−4.07	7.42	−1.57	1.02	−2.13	1.57	3.28	9.34
7	Palmatine	0.5	1.39	6.67	4.72	2.62	2.33	5.36	−2.58	5.71
		10	−2.96	4.02	−1.39	5.33	0.83	2.31	3.58	2.19
		80	−1.07	4.94	−2.16	4.69	−2.04	8.06	4.01	11.64
8	Puerarin	5	4.45	12.43	3.13	3.97	3.21	7.33	1.59	6.28
		100	−1.81	5.79	−2.68	6.31	3.38	4.19	2.11	5.20
		800	−2.97	7.64	−3.75	9.18	−1.96	8.57	3.51	7.29
9	Ononin	1.25	1.33	5.37	4.00	4.84	1.33	2.83	1.26	4.43
		25	−2.84	2.70	−2.94	6.04	−1.81	8.44	−1.57	4.91
		200	−2.71	7.91	−5.66	6.39	−2.99	8.46	5.05	4.78
10	Calycosin-7-O-*β*-D-Glc	0.5	−1.71	2.11	2.93	3.98	5.15	10.04	−4.06	5.75
		10	−5.46	9.60	−4.49	13.92	−4.79	2.77	3.71	3.74
		80	−3.13	7.42	−4.41	8.13	−3.03	6.38	3.84	6.32
11	Tanshinol	25	−2.80	3.11	−5.33	8.80	−3.31	9.84	−7.67	10.91
		500	−3.08	8.89	2.34	1.27	−2.36	4.79	−4.47	4.05
		4000	−2.12	8.35	−5.65	11.94	−3.87	5.09	−5.03	6.53
12	Rosmarinic acid	1.25	−4.00	5.93	−4.83	9.42	−5.33	7.49	−1.33	5.97
		25	−2.50	9.19	−1.62	3.66	−3.27	5.46	−4.57	7.32
		200	2.92	7.10	5.33	10.16	3.32	6.77	−1.43	2.35
13	Alkanoic acid	5	−2.00	10.85	3.33	13.03	−2.67	9.79	−3.52	5.79
		100	−5.79	13.22	−2.14	2.89	−4.88	3.81	−3.33	5.67
		800	−2.05	7.84	−3.99	9.35	−1.36	7.32	−3.80	5.13
14	Salvianolic acid B	2.5	−1.33	9.40	−2.17	12.67	−2.70	9.33	−2.67	4.24
		50	−3.04	10.25	−1.30	5.32	−1.86	2.41	−5.06	7.65
		400	−2.68	6.47	−3.36	4.13	−3.72	3.40	−2.68	7.22

**Table 7:** PK parameters of the fourteen analytes after oral administration of dose-1 and dose-2 of YGJZF (mean ± SD, *n* = 6).

No.	Analyte	Group	*T*_max_ (h)	*C*_max_ (ng/mL)	*T*_1/2_ (h)	AUC_0−*t*_ (ng h/mL)	AUC_0−∞_ (ng h/mL)	MRT_0−*t*_ (h)	CL (L/kg/h)
1	Daidzein	Dose-1	7.00 ± 0.00	70.60 ± 13.24	21.73 ± 5.42	892.44 ± 140.57	957.28 ± 166.21	17.37 ± 1.25	1.11 ± 0.18
		Dose-2	11.57 ± 1.13^*∗∗*^	185.67 ± 17.55^*∗∗*^	18.99 ± 6.25	2929.33 ± 356.15^*∗∗*^	3062.05 ± 336.23^*∗∗*^	16.98 ± 0.80	0.74 ± 0.08^*∗∗*^
2	Fermononetin	Dose-1	0.50 ± 0.00	9.62 ± 0.75	5.55 ± 1.41	25.07 ± 5.53	30.65 ± 6.86	3.75 ± 0.23	3.82 ± 0.84
		Dose-2	0.50 ± 0.00	23.42 ± 4.38^*∗∗*^	7.35 ± 1.03	48.16 ± 6.59^*∗∗*^	64.12 ± 8.47^*∗∗*^	3.66 ± 0.21	3.81 ± 0.98
3	Apigenin	Dose-1	7.33 ± 0.82	2.27 ± 0.37	5.19 ± 1.18	20.08 ± 3.50	22.06 ± 4.61	9.54 ± 0.46	1.06 ± 0.22
		Dose-2	11.57 ± 1.13^*∗∗*^	5.43 ± 0.95^*∗∗*^	11.07 ± 2.53^*∗∗*^	73.03 ± 8.18^*∗∗*^	87.12 ± 12.08^*∗∗*^	12.54 ± 0.69^*∗∗*^	0.43 ± 0.22^*∗∗*^
4	Luteolin	Dose-1	6.00 ± 1.10	5.83 ± 0.36	5.91 ± 1.03	62.24 ± 11.28	67.85 ± 6.61	9.39 ± 0.35	0.83 ± 0.15
		Dose-2	11.14 ± 1.46^*∗∗*^	9.44 ± 1.91^*∗∗*^	9.12 ± 1.96^*∗∗*^	128.32 ± 22.28^*∗∗*^	136.04 ± 20.17^*∗∗*^	12.05 ± 0.58^*∗∗*^	0.59 ± 0.17^*∗*^
5	Berberine	Dose-1	0.33 ± 0.00	10.92 ± 1.53	28.57 ± 3.83	60.82 ± 14.40	98.58 ± 12.98	24.43 ± 3.17	68.22 ± 14.22
		Dose-2	0.17 ± 0.00	19.54 ± 4.16^*∗∗*^	29.84 ± 5.08	82.99 ± 17.38^*∗*^	136.71 ± 19.25^*∗∗*^	19.28 ± 1.80^*∗∗*^	108.48 ± 21.26^*∗∗*^
6	Jatrorrhizin	Dose-1	0.50 ± 0.00	2.80 ± 0.54	5.21 ± 1.92	5.76 ± 1.15	7.53 ± 0.88	2.97 ± 0.10	142.45 ± 14.22
		Dose-2	0.17 ± 0.00	5.96 ± 1.02^*∗∗*^	9.01 ± 2.40^*∗*^	9.39 ± 1.67^*∗∗*^	15.60 ± 2.56^*∗∗*^	2.96 ± 0.36	111.50 ± 18.20^*∗∗*^
7	Palmatine	Dose-1	0.50 ± 0.00	6.56 ± 1.44	5.18 ± 1.01	22.80 ± 5.20	27.80 ± 6.08	4.07 ± 0.19	64.39 ± 12.97
		Dose-2	0.17 ± 0.00	13.00 ± 2.51^*∗∗*^	9.58 ± 1.92^*∗∗*^	53.12 ± 4.17^*∗∗*^	59.16 ± 10.41^*∗∗*^	3.90 ± 0.29	67.30 ± 13.15
8	Puerarin	Dose-1	0.47 ± 0.07	700.35 ± 88.69	18.22 ± 2.70	3658.38 ± 441.21	3835.09 ± 486.41	14.68 ± 2.00	33.56 ± 4.30
		Dose-2	0.26 ± 0.09^*∗∗*^	1474.99 ± 224.25^*∗∗*^	21.54 ± 5.83	5930.15 ± 614.81^*∗∗*^	6364.14 ± 655.14^*∗∗*^	15.28 ± 1.27	26.81 ± 2.71^*∗∗*^
9	Ononin	Dose-1	0.17 ± 0.00	6.73 ± 0.61	2.65 ± 0.51	14.87 ± 2.08	17.46 ± 1.68	2.41 ± 0.23	67.12 ± 6.57
		Dose-2	0.17 ± 0.00	15.05 ± 3.46^*∗∗*^	4.69 ± 1.48	20.11 ± 3.53^*∗*^	28.77 ± 6.34^*∗∗*^	2.20 ± 0.28	76.29 ± 10.10
10	Calycosin-7-O-*β*-D-Glc	Dose-1	0.50 ± 0.00	3.30 ± 0.13	3.85 ± 1.68	5.08 ± 0.62	6.38 ± 0.75	2.06 ± 0.17	142.62 ± 16.25
		Dose-2	0.17 ± 0.00	9.01 ± 1.66^*∗∗*^	6.79 ± 2.28^*∗*^	8.47 ± 1.96^*∗∗*^	11.64 ± 2.04^*∗∗*^	1.57 ± 0.12^*∗∗*^	123.81 ± 24.03
11	Tanshinol	Dose-1	0.75 ± 0.00	182.69 ± 21.72	3.70 ± 0.72	437.71 ± 97.41	508.99 ± 105.81	2.59 ± 0.08	12.65 ± 2.76
		Dose-2	0.17 ± 0.00	637.19 ± 71.44^*∗∗*^	7.85 ± 2.62^*∗∗*^	1365.06 ± 292.73^*∗∗*^	2169.55 ± 523.01^*∗∗*^	2.93 ± 0.31^*∗*^	4.74 ± 1.23^*∗∗*^
12	Rosmarinic acid	Dose-1	0.50 ± 0.00	89.13 ± 13.69	1.61 ± 0.44	149.22 ± 22.98	152.56 ± 21.53	2.02 ± 0.19	63.35 ± 9.20
		Dose-2	0.17 ± 0.00	170.94 ± 37.20^*∗∗*^	5.39 ± 1.27^*∗∗*^	283.95 ± 66.86^*∗∗*^	319.74 ± 51.17^*∗∗*^	2.68 ± 0.24	30.60 ± 6.86^*∗∗*^
13	Alkanoic acid	Dose-1	1.00 ± 0.00	14.30 ± 1.73	4.92 ± 1.81	83.18 ± 10.47	103.70 ± 14.48	4.50 ± 0.22	14.65 ± 2.80
		Dose-2	0.50 ± 0.00	27.27 ± 3.89^*∗∗*^	9.06 ± 2.28^*∗∗*^	99.73 ± 19.49	150.73 ± 21.69^*∗*^	4.22 ± 0.19	10.03 ± 2.31^*∗*^
14	Salvianolic acid B	Dose-1	0.50 ± 0.00	56.12 ± 10.41	3.09 ± 0.72	95.12 ± 15.15	105.11 ± 14.37	2.31 ± 0.26	130.09 ± 17.78
		Dose-2	0.17 ± 0.00	86.06 ± 13.74^*∗*^	5.08 ± 1.43^*∗*^	96.72 ± 16.56	117.56 ± 23.28	2.10 ± 0.28	140.23 ± 27.20

^*∗*^ *P* < 0.05, ^*∗∗*^*P* < 0.01.

## Data Availability

The data used to support the findings of this study are available from the corresponding author upon request.

## Abbreviations

ALD: Alcoholic liver disease
AUC_0−∞_: Area under the plasma concentration-time curve from 0 to infinity
AUC_0−*t*_: Area under the plasma concentration-time curve from 0 to the last time
CL: clearance
*C*_max_: Maximum plasma concentration
ESI: Electrospray ionization
Glc: Glucoside
IS1: Liquiritigenin
IS2: Hesperidin
LLOQ: Lower limit of quantitation
MRM: Multiple reaction monitoring
MRT: Mean residence time
PK: Pharmacokinetics
QC: Quality control
RE: Relative error
RSD: Relative standard deviation
*T*_1/2_: Terminal half-life value
TCM: Traditional Chinese medicine
*T*_max_: Maximum concentration time
UPLC-MS/MS: Ultraperformance liquid chromatography coupled with mass spectrometry
YGJZF: Yigan Jiangzhi formula.

## Supplementary Materials

Supplementary Materials The supplementary material we submitted is the graphical abstract of this manuscript.
