# Supplementary material for: Pharmacokinetic Comparisons of Different Combinations of Yigan Jiangzhi Formula in Rats: Simultaneous Determination of Fourteen Components by UPLC-MS/MS
Source: J Anal Methods Chem. 2020 Mar 21;2020:9353975. doi: 10.1155/2020/9353975 (PMC7114774; doi:10.1155/2020/9353975)
Supplement: Supplementary Materials — The supplementary material we submitted is the graphical abstract of this manuscript. [file 9353975.f1.pdf]

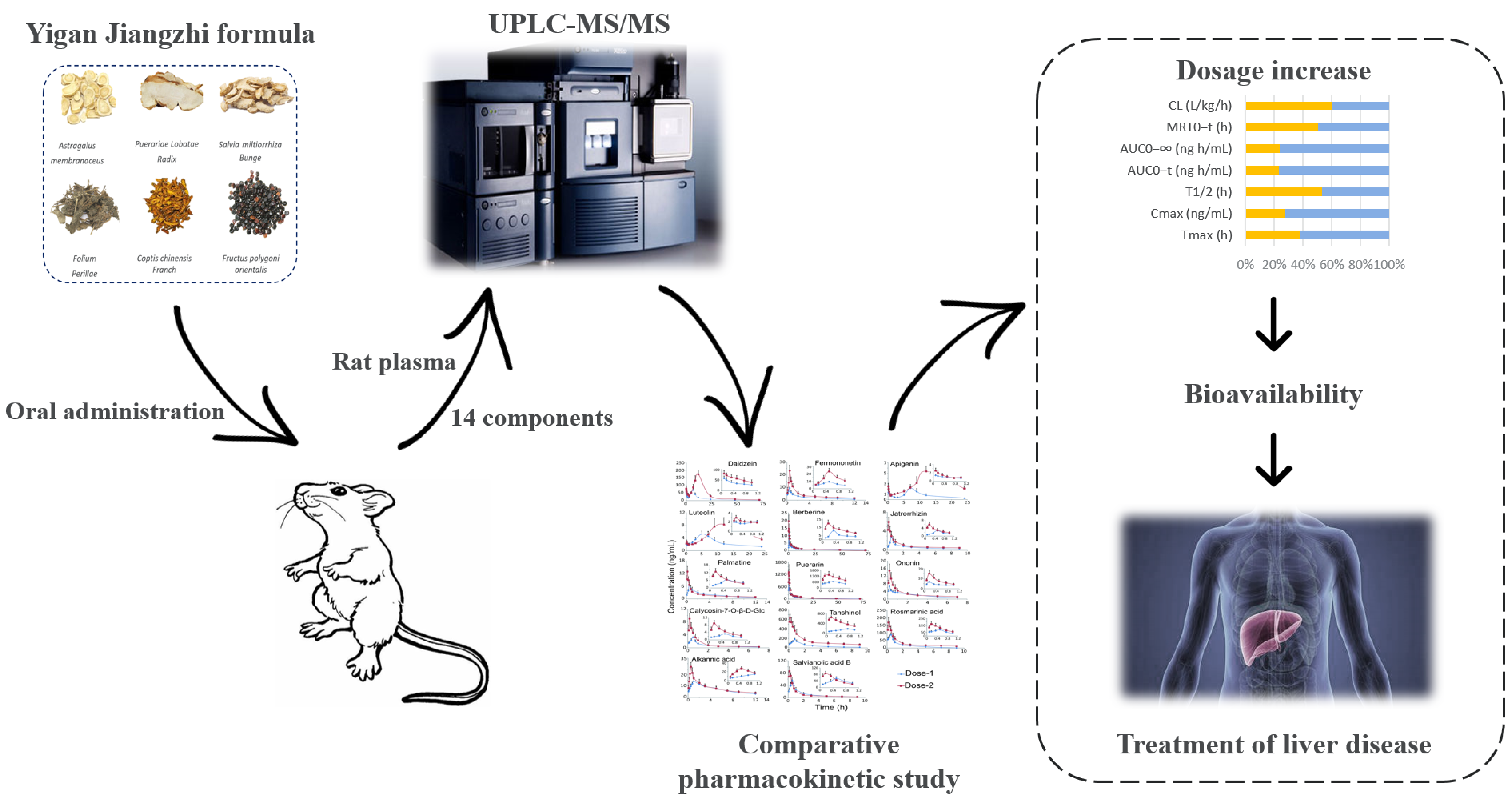

Yigan Jiangzhi formula
Astragalus membranaceus
Puerariae Lobatae Radix
Salvia miltiorrhiza Bunge
Folium Perillae
Coptis chinensis Franch
Fructus polygoni orientalis
UPLC-MS/MS
Oral administration
Rat plasma
14 components
Daidzein
Fermononetin
Apigenin
Luteolin
Berberine
Jatrorrhizin
Palmatine
Puerarin
Ononin
Calycosin-7-O-β-D-Glc
Tanshinol
Rosmarinic acid
Alkannic acid
Salvianolic acid B
Dose-1
Dose-2
Concentration (ng/mL)
Time (h)
Comparative pharmacokinetic study
Dosage increase
CL (L/kg/h)
MRT0–t (h)
AUC0–∞ (ng h/mL)
AUC0–t (ng h/mL)
T1/2 (h)
Cmax (ng/mL)
Tmax (h)
0%
20%
40%
60%
80%
100%
Bioavailability
Treatment of liver disease
